# Tympanic paraganglioma: two cases

**DOI:** 10.1016/S1808-8694(15)31140-X

**Published:** 2015-10-20

**Authors:** Romualdo Suzano Louzeiro Tiago, Fábio Marangoni Gil, Juparethan Trento Ribeiro, Patricya Santos Figueiredo dos Anjos, Patrícia Maria Sens, Lupércio Oliveira do Valle

**Affiliations:** 1 PhD in Sciences - Postgraduate Program in Otorhinolaryngology and Head and Neck Surgery - São Paulo Federal University. Assistant Physician at the Otorhinolaryngology Department - Hospital do Servidor Público Estadual de São Paulo and Hospital do Servidor Público Municipal de São Paulo; 2 Resident ENT - Otorhinolaryngology Department HSPM-SP; 3 Resident ENT - Otorhinolaryngology Department HSPM-SP; 4 Otorhinolaryngologist. Former Resident at the Otorhinolaryngology Department - HSPM-SP; 5 M.S. in Otorhinolaryngology - Postgraduate Program in Otorhinolaryngology - School of Medical Sciences - Santa Casa de São Paulo. ENT at the Otorhinolaryngology Department - HSPM-SP; 6 M.S. in Human Communication Disorders - PUC-SP. Assistant Physician - Department of Otorhinolaryngology HSPM-SP

**Keywords:** middle ear, temporal bone, paraganglioma

## INTRODUCTION

Paragangliomas originate from glomus bodies which are non-working temporal bone chemoreceptors, derived from neuroectodermal cells of the neural crest.^1^

Tympanic paragangliomas have pulsatile tinnitus as its most frequent symptom, followed by conductive hearing loss, otalgia and ear fullness.^2^ Some patients may present vertigo and otorhagia.^3^

Our goal is to present two cases of tympanic paragangliomas aiming at discussing the diagnostic and therapeutic aspects of such disorder.

## CASE REPORT

### Case 1

**Figure 1:**
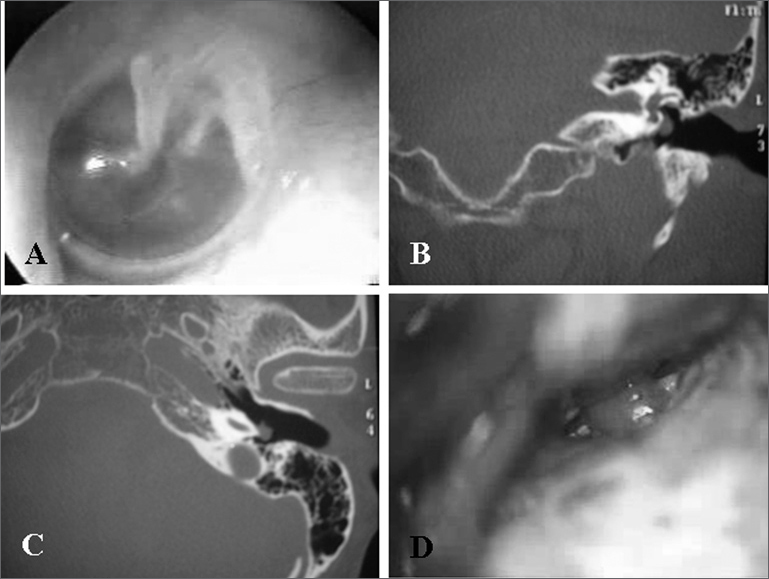
Tympanic paraganglioma (Case 1). Tumor touching the tympanic membrane (A); Temporal bone CT Scan, coronal (B) and axial (C) views, showing a soft tissue density mass on the left side, over the promontory and below the oval window; intraoperative photograph, retroauricular and transcanal approach showing the long process of the incus and the paraganglioma occupying part of the tympanic cavity(D).

RHL, 55 year old white female, came to us complaining of pulsatile tinnitus in her left ear, with two weeks of duration. During physical exam we noticed a red-wine-color lesion on the postero-inferior quadrant of the left tympanic membrane (Figure 1A). On CT scan we observed a soft tissue mass occupying part of the tympanic cavity, overlapping the promontory (Figures 1B and 1C). The patient was operated upon, through a retroauricular approach, which allowed us to clearly see the margins of the lesion and do a complete resection (Figure 1D). Pathologic exam confirmed the clinical diagnostic of paraganglioma. Audiometry was carried out on the third post-operative month, with auditory thresholds within normal ranges.

### Case 2

EPZ, 61 year old white female, came to us complaining of nasal obstruction and pruritus. During physical exam we noticed a red-wine-color retrotympanic lesion, over the promontory, in the left ear. We ordered a temporal bone CT scan, which showed a soft tissue mass restricted to the promontory on the left side. The patient was operated upon, through the same approach as described for the previous case. Pathology confirmed the clinical diagnosis of paraganglioma. We carried out an audiometric exam on the third post-operative month, with hearing thresholds within normal ranges.

## DISCUSSION

Tympanic paraganglioma is a benign neoplasia, more common to the middle ear^2^,^4^, and it is of great importance to suspect of such tumor in patients with pulsatile tinnitus and hearing loss. Jackson et al.^3^ evaluated a group of 60 patients with tympanic paraganglioma and observed that the major symptoms were: pulsatile tinnitus (77%), hearing loss (52%) and otalgia (18%). Approximately 5% of the patients diagnosed with tympanic paragangliomas are assymptomatic.3 Physical exam can lead us to suspect of tympanic paraganglioma when we see a reddish or red-wine-color lesion on the promontory, and in initial cases it is possible to outline the tumor in its entire circumference (Figure 1A). In larger lesions, that extend towards the hypotympanum, we have to use image diagnostic methods in order to differentiate tympanic paraganglioma from is jugular variety.^1^,^5^

CT scan is the method of choice to investigate tympanic paragangliomas, because of the very possibility of evaluating bone limits.1 Showing a clear border between the tumor and the jugular bulb helps identifies the lesion as tympanic paraganglioma (Figures 1B and 1C). Magnetic Resonance Imaging may be used to evaluate larger paragangliomas, in which we need to define tumor relations with soft tissue from the neck or with intracranial expansion.^1^

Myringotomy for biopsy purposes is not indicated when there is clinical suspicion of tympanic paraganglioma.^4^ Treatment may be broken down into palliative or definitive. Palliative treatment is reserved for elderly patients or those without clinical conditions to withstand surgery, in whom we use radiotherapy or clinical follow up only, since these are slow growth tumors and rarely present malignant transformation.3 The ultimate treatment, and the one of choice is surgery. The approach and surgery extension depend solely on lesion size. In those cases in which it is not possible to identify lesion borders, the retroauricular approach is recommended, with expansion to the facial nerve recess and hypotympanum.^2^, ^3^, ^4^ In the cases hereby reported, we used the retroauricular approach and small canaloplasty, just large enough to identify lesion borders (Figure 1D). After its identification, we detached the tumor from the promontory and cauterized the vascular pedicle, usually an offshoot from the ascending pharyngeal artery, with a bipolar electrocautery.

## FINAL REMARKS

Tympanic paragangliomas represent the most common middle ear benign neoplasia, and the early diagnosis of initial lesions much favors a reduced morbidity, which is high in the treatment of more extensive lesions.
